# Regional Variations in the Risk and Severity of Ciguatera Caused by Eating Moray Eels

**DOI:** 10.3390/toxins9070201

**Published:** 2017-06-26

**Authors:** Thomas Y. K. Chan

**Affiliations:** 1 Division of Clinical Pharmacology, Department of Medicine and Therapeutics, Faculty of Medicine, The Chinese University of Hong Kong, Prince of Wales Hospital, Shatin, New Territories, Hong Kong, China; tykchan@cuhk.edu.hk; Tel.: +852-3505-3907; 2 Centre for Food and Drug Safety, Faculty of Medicine, The Chinese University of Hong Kong, Hong Kong, China

**Keywords:** ciguatera, ciguatoxins, moray eels, *Gymnothorax* species

## Abstract

Moray eels (*Gymnothorax* species) from tropical waters have long been known to be high-risk species, and the consumption of particularly the viscera or ungutted eels can result in severe ciguatera (known as *Gymnothorax* or moray eel poisoning), characterized by prominent neurological features. In this review, the main objective was to describe the risk and severity of ciguatera caused by eating moray eels in different parts of the world. Moray eels can accumulate very high ciguatoxin (CTX) levels in the flesh and particularly the liver. Therefore, even the smaller ones can be toxic and the consumption of an average portion (particularly liver) can result in severe or fatal ciguatera. Moray eels (particularly when ungutted) must never be served in gatherings since they can cause mass poisoning because of their large sizes and high CTX levels. Apart from regulatory measures restricting or excluding access, the public should be repeatedly warned to avoid eating moray eels.

## 1. Introduction

Ciguatera is caused by eating reef fishes that have bioaccumulated ciguatoxins (CTX) via the marine food chain [1]. It is characterized by gastrointestinal, neurological, and, less frequently, cardiovascular signs and symptoms [1,2]. The ingestion of a large quantity and CTX-rich parts (head, skin, and viscera) and concomitant alcohol consumption will result in more severe poisoning and prolonged illness [2,3]. Ciguatera-related deaths are rare, but preventable, through public education and an early recognition of the warning features (e.g., coma and seizures) with immediate treatment [4].

CTX found in the Pacific Ocean (P-CTX), Caribbean (C-CTX), and Indian Ocean (I-CTX) regions differ in potency (P-CTX > I-CTX > C-CTX) [5]. As CTX are heat-stable and cannot be removed by cooking, avoiding and excluding high-risk fish are the best preventive measures. P-CTX-1 is the most potent CTX [5]. The lowest P-CTX-1 level associated with mild toxicity is estimated to be 0.1 μg/kg fish flesh and the consumption of ~1.0 μg/kg of P-CTX-1 will produce clear toxic symptoms [6]. Based on case studies in humans, a level of P-CTX-1 equivalents 0.01 μg/kg fish should not cause adverse effects, even in sensitive individuals, when eating a single fish meal [6].

Globally, fish species involved in ciguatera outbreaks are typically large, apex predators in tropical and subtropical coral reefs (e.g., moray eels, snappers, barracudas, groupers, Spanish mackerels, and humphead wrasse) [1,2,3,4,5,6,7,8]. The giant moray (*Gymnothorax javanicus*) and yellow-edged moray (*G. flavimarginatus*) should be specifically mentioned because of their high CTX levels and large sizes (total length of up to 300 cm and 240 cm or more, respectively) and hence the potential to cause mass ciguatera poisoning and even deaths [4,9]. Additionally, because of their broad distribution, moray eels (*Gymnothorax* species) have accounted for numerous outbreaks in the Indo-Pacific and Atlantic Ocean regions [10].

In this review, the main objective was to describe the risk and severity of ciguatera caused by eating moray eels (*Gymnothorax* species) in different parts of the world.

## 2. Literature Search and Data Syntheses

To identify journal articles and other relevant publications, a systematic search of Medline (1946 to May 2017), China Journal Net (1994 to May 2017), Google Scholar, and Google was performed, using ciguatera, ciguatoxins, moray eels, and *Gymnothorax* as the keywords. Other reports and press releases were identified from the literature database of our Centre for Food and Drug Safety [1,2,3,4] and the official websites of health or food safety authorities in different countries.

The framework proposed for the systematic risk assessment of large predator reef fishes was adopted [1,2,3,4]. Accordingly, the relative abundance (prevalence) of toxic moray eels, the severity of toxicity based on CTX levels and bioassay results, and previous ciguatera outbreaks were analyzed. The exact *Gymnothorax* species involved was specified if such information was provided.

## 3. Prevalence of Toxic Species and Severity of Toxicity in Wild-Caught Moray Eels

All published reports on the prevalence of toxic species, CTX levels, and bioassay results in wild-caught moray eels are summarized in Table 1. These 26 reports originated from the Central [11,12,13,14,15,16], Northern [16,17,18,19,20,21,22,23,24,25], and Southern [18,26,27,28,29] Pacific and the Caribbean [30,31,32,33,34,35,36], covering the moray eels in tropical (Kiribati [11,12,13,14,15,16], Johnston Islands [17,18], Marshall Islands [19], Hawaiian Islands [18,20], the southern coast of China [16,21], French Polynesia [18,26,27,28,29], and French West Indies [30,31,32,33,34,35,36]), tropical/subtropical (Taiwan [22,23]), and subtropical (Okinawa Island, Ryukyu and Amami Islands of Japan [18,24,25]) waters. The methods used to detect and quantify CTX in moray eel flesh and viscera are listed in the footnotes of Table 1 and some of these methods are described further in the Discussion [6,37]. A report from the Marshall Islands [38] was excluded because of concerns about the CTX extraction method used and the failure of top predator reef fishes to show toxicity in mice.

In Kiribati, 100% of *G. javanicus*, *G. flavimarginatus*, and *G. undulatus* from Marakei and Tarawa were toxic since their flesh contained P-CTX-1, or its equivalents, >0.01 µg/kg [13,14,15]. *G. javanicus* was generally more toxic than *G. flavimarginatus*, as indicated by the median (1.05 vs. 0.65 µg/kg) and mean (2.34 vs. 0.77 µg/kg) P-CTX-1 levels in the flesh [14,15]. By far, the highest P-CTX-1 equivalent in the flesh (81.8 µg/kg) was seen in *G. undulatus* [13]. In moray eels (*Gymnothorax* species), the liver was 4- to 15-fold (mean 9-fold) more toxic than the flesh [13]. In a 14-kg moray eel, the liver contained P-CTX-1 equivalents 539 µg/kg [13]. Unlike an earlier study using pooled samples of viscera of a similar size [11], *Gymnothorax* species weighing 1.5–14 kg showed a very strong positive relationship between size and the P-CTX-1 equivalent toxicity of the flesh and liver [13].

In the Johnston Islands, the liver of all *G. javanicus* specimens from one area (not specified) was toxic (130–550 mouse units (MU) per g) [18]. While there was a general correlation between liver and flesh toxicity, the liver was toxic even if the mongoose bioassay rating of flesh toxicity was zero.

In the Marshall Islands, 100% of the viscera and livers of the *G. javanicus* specimens in Enewetak were toxic [19]. The flesh of some *G. javanicus* specimens was also toxic, but to a lesser extent.

In the Hawaiian Islands, the flesh and liver of a *G. undulatus* specimen from Kaneohe Bay were not toxic [18]. The flesh of moray eels from Oahu and Hawaii was toxic [20].

In China, the flesh of *G. favagineus*, *G. javanicus* (0.4 kg), *G. melanospilus*, and *G. undulates* (1.1 kg) from the southern coast was not toxic [21]. The flesh of five moray eels bought in the local market of Shenzhen did not contain P-CTX [16].

In Taiwan, *G. pescardoris* was toxic, especially during Novemer–March and in its viscera (>3 MU/g) [22]. The viscera from six specimens of each of the *G. favagineus*, *G. hepaticus*, *G. javanicus* (mean size 1.1 kg), and *G. reticularis* groups were non-toxic [23].

In Japan, the flesh of *G. meleagris*, *G. flavimarginatus*, *G. pictus*, *G. undulates*, and two other moray eels (*Gymnothorax* species) from Okinawa Island was not toxic [18,24]. However, the liver of *G. undulates* and *G. flavimarginatus* was toxic (2 and 15 MU/g, respectively). The flesh of *G. meleagris* (5.8 kg) and *G. undulates* (12 kg) of the Ryukyu and Amami Islands was not toxic, but their liver was mildly toxic [25]. The flesh of two (out of three) *G. flavimarginatus* (7–8.4 kg) specimens from the Ryukyu and Amami Islands was mildly toxic, while the liver of a 7-kg eel with toxic flesh was moderately toxic [25].

In French Polynesia, the liver of *G. javanicus* in Tahiti (Popote Bay and Teavaraa Pass) and Bora Bora was toxic (80–1500 MU/g), and its flesh might be toxic (mongoose ratings 2–4) [18,26,27].

In the French West Indies, *G. funebris* and *G. moringa* were classified as high risk species in St. Martin and Anguilla [34,35]. *G. funebris* and *G. moringa* of St. Barthelemy were toxic and the CTX levels were much higher in the liver and viscera [30,31]. In *G. funebris* (size 3–15 kg), the ratio of the viscera to flesh CTX level was 3.5–10.4 (mean 6.9) and the ratio of the liver to flesh CTX level was 13.5–114.1 (43.7) [30]. In *G. moringa* (size 1.5–2 kg), the ratio of the viscera to flesh CTX level was 8.4–13.3 (10.9) [30]. Others reported a much lower frequency for the toxicity in *G. funebris* flesh, without mentioning the size of the specimens [32].

## 4. Worldwide Reports of Ciguatera Outbreaks

All published reports of ciguatera outbreaks caused by eating moray eels are summarized in Table 2, according to the geographical origins, i.e., the Central [39], Northern [23,40,41,42,43,44,45,46], and Southern [47,48,49,50,51] Pacific and the Caribbean [52]. The 15 reports involved the moray eels from tropical (Kiribati [39], Marshall Islands [40], Northern Mariana Islands [41], southern coastal cities of China [42,43,44,45], French Polynesia [47,48], Samoa [49,50,51], and Antigua [52]) and subtropical (Taiwan [23] and the Ryukyu and Amami Islands of Japan [46]) waters. Four of these 15 reports were confirmed cases in New Zealand [49,50,51] and the UK [52] after the consumption of moray eels from Samoa and Antigua.

In Kiribati, there were two outbreaks. In 1947, a big black ungutted moray eel from Phoenix Islands was cooked and shared by six people [39]. Five men who ate the flesh were asymptomatic, but the man who ate the fatty belly developed severe ciguatera lasting over three weeks. In 1961, two men developed a comatose state and died on the same night or one week later after eating part of a large moray eel from Tarawa [39]. It was cooked without gutting or cleaning.

In the Marshall Islands, an outbreak of severe ciguatera occurred in 1953 after the consumption of a cooked moray eel (likely *G. flavimarginatus*) caught in Kwajalein [40]. One of the six affected subjects was particularly ill with seizures and a comatose state, and died 25 days later.

In the Northern Mariana Islands, a large *G. flavimarginatus* specimen caught in Saipan was responsible for a large outbreak of severe ciguatera in 1949 [41]. All 57 people who ate the head and half of this eel, including the skin and broth, were ill. Coma (25%) and seizures (19%) were common. There were two deaths.

In the southern coastal cities of China, there were four reports. In 1999, nine people were treated in a hospital in Guangzhou during January–April after eating moray eels [42]. A female who ate the intestines had the most severe symptoms. In 1999–2002, a moray eel accounted for one of the five ciguatera outbreaks requiring admissions to a hospital in Shenzhen [43]. In 2004, a female and her four relatives were hospitalized in Dongguan with ciguatera after eating moray eel [44]. She then returned to Hong Kong for further treatment. In 2005, moray eels bought in a local market on the same day were responsible for two outbreaks in Hong Kong, each affecting two to three subjects [44].

In Taiwan, the only report involved a male, who ate *G. javanicus* flesh at a fishing port in Taipei in 2004.

In Japan, 10 outbreaks affecting ~95 people after eating *G. flavimarginatus* were reported from the Ryukyu and Amami Islands, from before 1930 to 1968.

In French Polynesia, two men developed severe ciguatera illness after eating moray eels with neurological features highly suggestive of Guillain-Barré syndrome and acute inflammatory polyneuropathy. In 1979, a 32-year-old male chronic alcoholic living in the Australes Islands was severely poisoned, especially after eating the liver, whereas moderate symptoms were seen in others eating eel flesh only [47]. The second case, reported in 2009, involved a 43-year-old male chronic alcoholic living in the Toumotu Islands [48].

In New Zealand, there were three ciguatera outbreaks, which were all related to the consumption of moray eels from Samoa. According to the national notifiable disease surveillance database, two outbreaks, each involving two adults, occurred in 1999 and 2003 [49]. The latest outbreak in 2016 involved four adults due to the consumption of moray eel flesh containing CTX-1B 2.74 µg/kg [50,51].

In the UK, a 46-year-old man was hospitalized in 1979 after eating *G. goringa* and greater amberjack brought back from Antigua [52].

## 5. Discussion

Toxic dinoflagellates of the *Gambierdiscus* species in the tropical and subtropical waters produce CTX precursors [9]. On passing up the food chain, CTX precursors and intermediate congeners are metabolized and bioaccumulated, such that moray eels and other apex predators should contain the greatest amount and the most potent form of CTX, especially in the viscera [1,2,3,4]. There are major regional differences in the composition and inherent toxicities of the *Gambierdiscus* species [53]. The CTX profiles and levels even in the same fish species vary with the geographical origins [1].

Methods to detect and quantify CTX in fish are required in an investigation of ciguatera outbreaks, the identification of toxic species, and the formulation of risk management strategies. The methods involved, advantages, and disadvantages were described elsewhere [6,37]. A mouse bioassay provides a measure of the total toxicity, but it is not sensitive enough -the detection limit for P-CTX-1 ~0.56 µg/kg [6]. There are three different biomolecular methods-cytotoxicity assays, receptor-binding assays, and immunoassays. A mouse neuroblastoma assay [13] measures the activity of P-CTX-1 (detection limit 0.0016 µg/kg) and other CTX. LC-MS/MS and HPLC-MS/MS are very sensitive chemical methods (detection limits for P-CTX-1, P-CTX-2, and P-CTX-3 = 0.0005, 0.005, and 0.005 versus 0.0009, 0.019, and 0.017 µg/kg) [14,15,16]. LC-MS/MS is very specific, but more expensive. Challenges in CTX analyses include low toxin levels and the diversity of congeners present in a fish sample [7]. For practical applications, a two-tiered approach is adopted. For example, the FDA proposes the mouse neuroblastoma assay for screening, coupled with LC-MS/MS for the molecular confirmation of CTX in a fish sample [7].

Moray eels (*Gymnothorax* species) from tropical waters (Table 1) have long been known to be high-risk species, and the consumption of particularly the viscera or ungutted eels can result in severe ciguatera (known as *Gymnothorax* or moray eel poisoning), characterized by prominent neurological features [39,54]. In a recent review of worldwide reports on ciguatera-related deaths, moray eels feature prominently [4]. The risk and severity of ciguatera after eating large, apex predators are known to vary with their geographical origins [1]. A systematic analysis of such information is particularly indicated for moray eels because of their potential to cause mass poisoning and deaths [4,9]. Accordingly, published reports on the prevalence (P-CTX-1 equivalents >0.01 μg/kg) and levels of toxicity in the flesh and viscera and the severity of ciguatera illness after the consumption of moray eels from both tropical and subtropical waters were reviewed.

Based on the limited information available (Table 1), several observations could be made about the risk and levels of ciguatoxicity in moray eels from tropical and subtropical regions. In Kiribati, 100% of *G. javanicus*, *G. flavimarginatus*, and *G. undulatus* were toxic, including the smaller specimens (*G. javanicus* 1.1–3.5 kg and *G. flavimarginatus* 0.6–1.4 kg) [13,14,15]. The level of toxicity in the flesh was related to the origin (Tarawa > Marakei), species (*G. javanicus* and *G. undulatus* > *G. flavimarginatus*) and, in particular, the size of the specimens [13,14,15]. The most toxic eel that has been identified thus far is a *G. undulatus* containing P-CTX-1 equivalents 81.8 µg/kg [13]. In the French West Indies, some [30,31,32,33], but not all [32], reports indicated that 100% of *G. funebris* and *G. moringa* were toxic. In the Marshall Islands and French Polynesia, some *G. javanicus* were toxic [18,19,26]. In the Ryukyu and Amami Islands, some *G. flavimarginatus* exhibited a mild toxicity [25]. It is important to remember that the liver of moray eels could be toxic, even if their flesh presents non-toxic results. This has been shown in the Johnston Islands [17,18], Marshall Islands [19], Okinawa Island [18], Ryukyu and Amami Islands [25], and French Polynesia [18,26]. In fact, P-CTX was much more concentrated in the liver than the flesh of moray eels. In Kiribati, the liver of moray eels in Marakei was on average nine times more toxic than the flesh (range 4.1–15.4 fold) and there was a positive correlation between liver toxicity and eel size [13]. The liver of a 14-kg moray eel contained P-CTX-1 equivalents 539 µg/kg [13]. In the French West Indies, the ratio of the viscera to flesh and liver to flesh CTX level in *G. funebris* from St. Barthelemy was 3.5–10.4 (mean 6.9) and 13.5–114.1 (mean 43.7), respectively [30]. As for *G. moringa*, the ratio of the viscera to flesh CTX level was 8.4–13.3 [30]. In the Johnston Islands, the liver of all *G. javanicus* specimens from one area was toxic (130–550 MU/g) [18]. In the Marshall Islands, the viscera and liver of *G. javanicus* from Enewetak were toxic [19]. In French Polynesia, the liver of *G. javanicus* was toxic (70–1000 MU/g) [18,26,27].

Detailed analyses of previous ciguatera outbreaks can also provide useful information on the severity of ciguatoxicity in moray eels from different regions (Table 2). In particular, any occurrence of mass poisoning, deaths, and severe ciguatera (characterized by coma, seizures, focal neurological signs, etc.) [4] should be realized. Fatal ciguatera related to moray eels has occurred in Kiribati [39], the Marshall Islands (*G. flavimarginatus*) [40], and the Northern Mariana Islands (*G. flavimarginatus*) [41]. Mass poisoning affecting 57 people (including 14 in a comatose state and two deaths) was caused by eating the head and half of a *G. flavimarginatus* in the Northern Mariana Islands [41]. Non-fatal ciguatera dominated by severe neurologic features occurred in Kiribati [39], the Marshall Islands [40], the Northern Mariana Islands [41], and French Polynesia [47,48]. Ungutted moray eels or CTX-rich parts have been involved in some outbreaks [39,41,42,47]. In two chronic alcoholics with severe Guillain-Barré syndrome and acute inflammatory polyneuropathy [47], it was not stated if the moray eel was consumed together with alcohol, which could aggravate the symptoms [2].

Based on the reported information in Table 1 and Table 2, it should be easy to appreciate the risk and severity of ciguatera caused by eating moray eels. Firstly, the lowest P-CTX-1 level in fish associated with mild toxicity in humans is 0.1 μg/kg [6]. In Kiribati, the flesh of a *G. undulatus* (81.8 μg/kg) and the liver of a 14-kg moray eel (539 μg/kg) contained 818 and 5390 times this level, respectively [13]. In French Polynesia, the liver of a *G. javanicus* (1000 MU/g = 5000 μg/kg P-CTX-1) contained 50,000 times as much [27]. Secondly, the ingestion of 0.5 μg P-CTX-1 (0.01 μg/kg for a 50-kg subject) in one meal will produce obvious toxicity in most people [6,11]. In Kiribati, if its flesh contained 40 μg/kg P-CTX-1, a gutted 14-kg moray eel would have the potential to poison up to 1120 people [13].

Some experts recommend the complete avoidance of moray eels as they are commonly highly toxic (unless when coming from districts without a history of ciguatera) [55]. Across the Pacific, many strategies are adopted to reduce the risk, including a general avoidance of highly risky species (e.g., *G. javanicus*) and discarding their viscera (particularly the liver) [56]. In French Polynesia, regulations prohibit the sale of all moray eels [57]. In New Caledonia, *G. javanicus* is considered by some experts as highly risky, regardless of its size, and should be banned [58]. In Australia, the largest center of fish distribution (Sydney Fish Market) rejected *G. javanicus* and fish from high-risk regions (Kiribati and Marshall Islands) [59]. In the U.S. Virgin Islands (Caribbean), based on interviews and information from fishermen, *G. funebris* and *G. moringa* were given a risk ranking of two (frequent poisoners) [60]. In Mauritius (Indian Ocean), the sales of *G. javanicus* and 16 other fish species have been banned [57]. It was a complex choice for consumers between species desirable and those safe for eating.

Apart from regulatory measures restricting or excluding access, the public must be warned that moray eels can accumulate very high CTX levels in the flesh and particularly the liver. Therefore, even the consumption of an average portion can result in severe or fatal ciguatera (Table 2). Depending on their regions of origin, even the smaller moray eels (0.6–3.5 kg) can be toxic [13,14,15]. Moray eels (particularly ungutted) must never be served in gatherings since they can cause mass poisoning because of their large size and high CTX levels [41].

## 6. Conclusions

Moray eels (*Gymnothorax* species) from tropical waters have long been known to be high-risk species. Based on the reports on the frequency and levels of ciguatoxicity in wild-caught samples and previous outbreaks, the risk and severity of ciguatera illness caused by eating moray eels in different parts of the world were systematically analyzed. Moray eels can accumulate very high CTX levels in the flesh and viscera (particularly the liver). Therefore, even the smaller specimens can be toxic and the consumption of an average portion (particularly the liver) can result in severe or fatal ciguatera. Moray eels (particularly ungutted) should never be served in gatherings since they can cause mass poisoning. Apart from regulatory measures restricting or excluding access, the public should be repeatedly warned to avoid eating moray eels.

## Figures and Tables

**Table 1:** Toxic species prevalence and the severity of toxicity in wild-caught moray eels in the world.

Place (Year *)	Details
**Kiribati**	
Tarawa (1987–1989) [11]	217 *G. javanicus* (subsample of 38 eels, size 0.6–10 kg, mean 3.6 kg) from 9 collections over a 500-day period; viscera of similar size (*n* = 1–18) pooled into 47 samples; their toxicity ranged from 0.59 to 7.3 (mean 2.43) MU per g; exponential decline in toxicity (t_½_ 264 days) over 500 days; no significant relation between toxicity and mean viscera weight (*n* = 47).
Tarawa (1991) [12]	In viscera of *G. javanicus*, P-CTX-1, P-CTX-2 and P-CTX-3 were the major CTX present; their relative yields by weight = 1:0.6:0.2; their relative yields by total toxicity to mice = 1:0.06:0.06.
Marakei & Tarawa (2009) [13]	100% of *G. flavimarginatus* (*n* = 13) and 100% of undulated moray (*G. undulatus*) (*n* = 19) were toxic ^†^ (P-CTX-1 equivalents >0.01 µg/kg in flesh); moray eels and groupers generally more toxic than other fishes by an order of magnitude; the 3 most toxic fishes were all *G. undulatus* (P-CTX-1 equivalents 81.8, 27.9 and 17.2 µg/kg); P-CTX-1 equivalents higher in *G. undulatus* than *G. flavimarginatus* (mean 7.45 vs. 3.02 µg/kg) and in Tarawa than Marakei eels (0.17–81.8 vs. 0.02–0.40 µg/kg). In 6 moray eels (*Gymnothorax* species, size 1.5–14.0 kg) from Marakei, liver on average was 9 times more toxic than flesh (range 4.1–15.4 fold); both liver and flesh showed a very strong +ve relationship between size and P-CTX-1 equivalent toxicity; toxicity in liver of a 14-kg eel = 539 µg/kg, i.e., 50,000 times higher the safety level.
Marakei (2013) [14]	100% of *G. javanicus* (*n* = 20, size 1.1–13.2 kg, median 5.5 kg) were toxic ^‡^, with P-CTX-1, P-CTX-2, P-CTX-3 and total P-CTX levels in their flesh of 0.03–17.0 (median 1.05), 0.02–14.9 (0.34), <0.005–4.72 (0.11) and 0.05–34.5 (1.32) µg/kg, respectively. 100% of *G. flavimarginatus* (*n* = 15, size 0.6–2.8 kg, median 1.5 kg) were toxic, with P-CTX-1, P-CTX-2, P-CTX-3 and total P-CTX levels in their flesh of 0.05–39.2 (median 0.65), <0.01–24.4 (0.32), <0.01–5.94 (0.08) and 0.06–69.5 (0.91) µg/kg, respectively.
Marakei (2013) [15]	100% of *G. javanicus* (*n* = 8, size 3.5–12.6 kg) were toxic ^‡^, with P-CTX-1, P-CTX-2, P-CTX-3 and total P-CTX levels in their flesh of 0.03–7.34 (mean 2.34), 0.02–14.9 (4.71), <0.005–4.72 (1.01) and 0.05–27.0 (7.93) µg/kg, respectively. 100% of *G. flavimarginatus* (*n* = 6, size 1.4–2.8 kg) were toxic, with P-CTX-1, P-CTX-2, P-CTX-3 and total P-CTX levels in their flesh of 0.21–2.43 (mean 0.77), 0.09–3.48 (0.81), 0.02–0.48 (0.15) and 0.37–6.39 (1.73) µg/kg, respectively.
(2014) [16]	In a moray eel flesh sample, the amounts ^§^ of P-CTX-1, P-CTX-2 and P-CTX-3 were 0.84, 1.00 and 0.55 μg/kg, respectively.
**Johnston Islands**	
(1968) [17]	Liver of *G. javanicus* with mongoose bioassay ratings (0 = non-toxic, 5 = death) of 4 and 5 contained distinctly large amounts of CTX.
(1969) [18]	Liver of all *G. javanicus* from one area was toxic (130–550 MU/g); there was some correlation between liver toxicity and flesh toxicity, but liver was toxic even if mongoose rating of flesh was 0.
**Marshall Islands**	
Enewetak (1980) [19]	100% of viscera and liver of *G. javanicus* (*n* = 9, size 3.6–13.0 kg) were toxic, with mongoose bioassay ratings of 5 (*n* = 3), 4 (*n* = 3), 3 (*n* = 1), 2 (*n* = 2); flesh from 2 eels of rating 4 toxic (rating 1 or 2); flesh from 1 eel of level 5 also toxic (rating 3).
**Hawaiian Islands**	
Kaneohe Bay (1969) [18]	Flesh (mongoose bioassay) and liver (mouse bioassay) of a *G. undulatus* were non-toxic.
Oahu & Hawaii (1985) [20]	Flesh from moray eels was toxic (mouse bioassay rating 2 and enzyme immunoassay).
**China**	
Southern coast (2007) [21]	Flesh of *G. favagineus* (0.7 kg) *G. javanicus* (0.4 kg), *G. melanospilus* (2.1 kg) and *G. undulates* (1.1 kg) was not toxic (Cigua-Check^®^).
Shenzhen (2014) [16]	Flesh of 5 moray eels bought in local market did not contain P-CTX ^§^.
**Taiwan**	
Adjacent waters (1992–1994) [22]	*G. pescardoris* was toxic (mouse bioassay), especially in November to March and in viscera (>3 MU/g).
Different places (2009) [23]	Viscera from 6 specimens each of *G. favagineus* (mean 0.3 kg), *G. hepaticus* (0.3 kg). *G. javanicus* (1.1 kg) and *G. reticularis* (0.1 kg) were non-toxic (mouse bioassay).
**Japan**	
Okinawa Island (1965) [24]	Flesh of *G. meleagris* (1.1 kg), *G. pictus* (1 kg) and 2 other moray eels (*Gymnothorax* species, 0.9–1 kg) was not toxic (cat bioassay).
Okinawa Island (1969) [18]	Flesh of *G. meleagris*, *G. undulates* and *G. flavimarginatus* was all non-toxic (cat bioassay); however, their liver contained 0 (*G. meleagris*), 2 (*G. undulates*) and 15 (*G. flavimarginatus*) MU/g of toxicity.
Ryukyu & Amami Islands (1966–1967) [25]	Flesh of *G. meleagris* (5.8 kg) and *G. undulates* (12 kg) was not toxic (cat bioassay), but their liver was mildly toxic. Flesh of 2 (out of 3) *G. flavimarginatus* (7–8.4 kg) was mildly toxic (mouse bioassay); liver from the 7-kg moray eel with toxic flesh was moderately toxic.
**French Polynesia**	
Tahiti (1968) [18,26]	Liver of 3 *G. javanicus* from Popote Bay was toxic (80 MU/g = 1500 MU/g = 2); their flesh was also toxic (mongoose bioassay ratings 2–4). Liver of *G. javanicus* from Teavaraa Pass was toxic (70 MU/g), but its flesh was not (mongoose bioassay).
Tahiti (1976) [27]	Liver of *G. javanicus* from Popote Bay was toxic (1000 MU/g).
Bora Bora (1969) [18]	Liver of *G. javanicus* was toxic (120 MU/g), but its flesh was not (mongoose bioassay).
Papeete? (1996) [28]	2 flesh samples and 1 liver sample of moray eel donated for biotest contained CTX 0.8, 13 and 80 µg/kg, respectively.
Unpublished data (2000) [29]	P-CTX-1B (P-CTX-1) and its congeners contributed to at least 51%, and P-CTX 3C to 15% of the overall toxicity in flesh.
**French West Indies**	
St. Barthelemy (1980–1983) [30]	5 out of 5 *G. funebris* (size 3–15 kg) were toxic; flesh contained 0.29–0.92 MU/g (mean 0.51); ratio of viscera to flesh CTX level = 3.5–10.4 (6.9); ratio of liver to flesh CTX level = 13.5–114.1 (43.7). 2 out of 2 *G. moringa* (size 1.5–2 kg) were toxic; flesh contained 0.12–0.30 MU/g (mean 0.21); ratio of viscera to flesh CTX level = 8.4–13.3 (10.9).
St. Barthelemy (1980–1984) [31]	6 out of 6 *G. funebris* (size 3.5–14.5 kg) were toxic; the flesh contained CTX 0.05–0.49 (*n* = 4) or 0.05–0.99 (*n* = 2) MU/g.
St. Barthelemy (1979–1985) [32]	2 out of 24 *G. funebris* were toxic, with CTX 0.5–1.0 MU/g in flesh. 2 out of 2 *G. moringa* were toxic, with CTX 0.05–0.5 MU/g in flesh.
St. Barthelemy (1982) [33]	Flesh of *G. funebris* (*n* = 2, size 4–6 kg), and the great barracuda, had an especially high content of fast-acting CTX (mouse bioassay).
St. Martin & Anguilla (1985–1992) [34,35]	Based on the mosquito biotest results and the epidemiology of ciguatera in 1985–1987 and 1991–1992, both *G. funebris* and *G. moringa* were classified as high risk species.
St. Barthelemy & Guadeloupe (1993–1999) [36]	Liver of *G. funebris* contained CTX at a significant level (chick bioassay) even if their flesh was tested negative for toxicity (mouse bioassay).

* The year of study or (if this was not stated) the year of publication of the report. One mouse unit (MU) = the LD_50_ dose for a 20-g mouse = 5 ng of P-CTX-1 (CTX-1) [7]. ^†^ Mouse neuroblastoma assay measured the activity of P-CTX-1 and other CTX. ^‡^ Liquid chromatography-tandem mass spectrometry (LC-MS/MS). ^§^ High performance liquid chromatography with electrospray-tandem mass spectrometry (HPLC-MS/MS).

**Table 2:** Worldwide reports of ciguatera outbreaks caused by eating moray eels.

Place (Year *)	Details
**Kiribati**	
Phoenix Islands (1947) [39]	6 men ate a big ungutted black moray eel. 5 men who ate the flesh only had no symptoms. 1 man who ate the fatty belly felt very ill 0.5 h later; felt very cold in the wind and, after moving away, then very hot; severe dizziness with loss of balance, severe stomach pain, violent vomiting, diarrhea and lower limb weakness; lied in bed in week 1; intense itchiness in week 2, but felt better with improvement in balance; very much better by week 3 with generalized skin peeling.
Tarawa (1961) [39]	2 men became very ill with violent vomiting and severe stomach ache after eating part of a large ungutted moray eel; the old man was in coma and died in hospital in the same night; the young man stayed in hospital for 1 week with intense itchiness and skin peeling off, followed by coma and death.
**Marshall Islands**	
Kwajalein (1953) [40]	All 6 people who ate a cooked eel (likely *G. flavimarginatus*) developed marked neurological features in 2 h (*n* = 5) or 7 h (*n* = 1), including anxiety, panic, dizziness, pronounced generalized malaise, marked difficulty in voluntary movements of facial muscles and the extremities, generalized numbness, hyporeflexia, carpopedal spasm, Chvostek’s sign, Romberg’s sign, and severe ataxia. Some had severe retching with vomiting. One patient was particularly ill, with the additonal features of grand mal seizures, deep coma, generalized areflexia, no responses to pain stimuli, spastic upper limbs but weakness and hypotonicity of all other muscles, fever and Cheyne-Stokes breathing; ventilated; coma for 24 days, died 25 days after eel ingestion.
**Northern Mariana Islands**	
Saipan (1949) [41]	All 57 people who ate the head and half of a yellow-edge moray eel (*G. flavimarginatus*) (6 feet long, 1 foot thick) and the broth were poisoned. All 57 people had tingling/numbness of the mouth, hands and feet. Some experienced syncope, and about 20 were shouting. 50 people developed vomiting. 50 people were unable to talk. After initial assessment in a local health facility, all 57 people stayed in bed for the next 2 days. 17 people were hospitalized from day 4, with coma (*n* =14), ↓ function of respiratory chest muscles (*n* = 13), profuse sweating (*n* = 12), seizures (*n* = 11), tachypnea (*n* = 10), trismus (*n* = 8), hyperthermia (*n* = 7), conjugate deviation of eyes to the right (*n* = 6), purposeless movements, areflexia, ataxia, dizziness, blurred vision, ↑ bronchial secretions and neuropathy. 2 died from bilateral bronchopneumonia after 14–18 days of coma.
**China**	
Guangzhou (1999) [42]	9 people (4M, 5F, aged 5–80 y), including a family of 3, were admitted to hospital during January–April after eating the flesh or viscera (liver, intestines) of moray eel. Symptoms appeared after 1–4 h and lasted 3–14 days (mean 6 days). All had nausea, vomiting, abdominal pain, diarrhea, and numbness of the tongue, lips and 4 limbs. Some had reversal of hot-cold sensation (*n* = 6), dizziness/headache (*n* = 5), insomnia (*n* = 5), sinus bradycardia (*n* = 5) or hypotension (*n* = 4). F/48 who ate the intestines had the most severe symptoms, including dyspnea, tachypnea (28 breaths/minute) and shock (BP 81/51 mmHg).
Shenzhen (2004) [43]	18 people (10M, 8F, aged 4–60 y) from 5 outbreaks (1 caused by moray eel) were admitted to a hospital in 1999–2002, with mild to moderate symptoms, which resolved on charge or within 2–3 weeks.
Dongguan (2004) [44]	F/33 and 4 of her relatives in China with ciguatera after eating moray eel in Dongguan required admission to a local hospital; the woman returned to Hong Kong 3 days later and was hospitalized for 1 more day.
Hong Kong (2005) [45]	2 outbreaks of ciguatera caused by eating moray eel bought in a local fish market on the same day. 1st outbreak—3F aged 12–50 y, with face, tongue and limb numbness, diarrhea, vomiting and abdominal pain 5–7.5 h later; no hospitalization. 2nd outbreak—M/43 and F/44, with numbness, diarrhea, nausea and abdominal pain ~2 h later; discharged after hospital treatment.
**Taiwan**	
Taipei (2004) [23]	47-year-old subject complained of pricking of the lips, tongue and throat, vomiting, abdominal cramps, diarrhea, headache, dizziness, vertigo and paralysis after eating moray eel (*G. javanicus*) flesh.
**Japan**	
Ryukyu & Amami Islands (1969) [46]	10 outbreaks affecting ~95 people after eating *G. flavimarginatus* (flesh only in 8 outbreaks, parts eaten unknown in 2 outbreaks), 1930 to 1968. Symptoms included aching joints (100%), fatigue (70%), itching (50%), diarrhea (50%), loss of appetite (20%), headache (20%), flushing (20%), and perioral/limb numbness (20%).
**French Polynesia**	
Austral Islands (1979) [47]	A group of people ate a moray eel; moderate symptoms if only the flesh was eaten; severe, prolonged illness in M/32, with chronic alcoholism, who ate especially the liver; gastroenteritis symptoms, pruritus and dysesthesia a few hours later; then paraplegia progressing to tetraplegia with proximal predominance and breathing difficulties; ICU care; cerebrospinal fluid showed ↑ protein only; electromyography (EMG) showed acute polyneuropathy; full recovery 10 months later.
Toumotu Islands (2009) [48]	M/43, with chronic alcoholism, ate a moray eel meal; asthenia, diarrhea, abdominal pain and dizziness; then ascending tetraparesis, areflexia and dyspnea; ICU care; cerebrospinal fluid showed ↑ protein only; EMG showed acute polyneuropathy, with improvement 2 months later.
**New Zealand**	
(1999) [49]	2 people aged 57–65 y were ill 3 h after eating moray eel from Samoa.
(2003) [49]	2 people aged 43–56 y developed diarrhea, abdominal pain, vomiting, chills, vertigo, difficulty in walking, loss of energy and paresthesia of hands, 1 h after eating part of a 5–8 kg moray eel privately imported from Samoa; other symptoms reported included headache, paresthesia of lips, numbness in hands/ legs, depression, joint pain, visual defects, short-term memory loss; symptoms lasted <1 day to 25 days.
Wellington (2016) [50,51]	Family of 3 (M/67, F/58, M/41) and their neighbor (F/67) hospitalized after eating cooked moray eel flesh bought in a market in Samoa. Eel flesh contained CTX-1B 2.74 μg/kg. Thus, eating about 36 g of the flesh could result in toxicity. All had nausea, vomiting, diarrhea, hypotension, bradycardia, fever, and paresthesia around mouth and extremities. Severe cardiotoxicity (SBP 61–72, HR 30–32 bpm) in F/67, M/67, M/41, requiring admission to high dependency unit and 4–6 hourly atropine. Cardiotoxicity for 3–4 days; lethargy/fatigue lasting a few weeks in all.
**UK**	
London (1979) [52]	M/46 of West Indian ethnicity was hospitalized after eating portions of dried and salted moray eel (*G. goringa*) and greater amberjack prepared by himself and brought back from Antigua. Symptoms appeared within 0.5 h, including nausea, diarrhea, abdominal pain, hypotension, and bradycardia (which persisted for >7 weeks).

* The year of study or (if this was not stated) the year of publication of the report. SBP = systolic blood pressure; HR = heart rate.
